# A Metastable p-Type Semiconductor as a Defect-Tolerant Photoelectrode

**DOI:** 10.3390/molecules26226830

**Published:** 2021-11-12

**Authors:** Zahirul Sohag, Shaun O’Donnell, Lindsay Fuoco, Paul A. Maggard

**Affiliations:** Department of Chemistry, North Carolina State University, Raleigh, NC 27609-8204, USA; zisohag@ncsu.edu (Z.S.); scodonne@ncsu.edu (S.O.); lfuoco@harris.com (L.F.)

**Keywords:** metastability, photoelectrode, solar energy conversion, semiconductor

## Abstract

A p-type Cu_3_Ta_7_O_19_ semiconductor was synthesized using a CuCl flux-based approach and investigated for its crystalline structure and photoelectrochemical properties. The semiconductor was found to be metastable, i.e., thermodynamically unstable, and to slowly oxidize at its surfaces upon heating in air, yielding CuO as nano-sized islands. However, the bulk crystalline structure was maintained, with up to 50% Cu(I)-vacancies and a concomitant oxidation of the Cu(I) to Cu(II) cations within the structure. Thermogravimetric and magnetic susceptibility measurements showed the formation of increasing amounts of Cu(II) cations, according to the following reaction: Cu_3_Ta_7_O_19_ + x/2 O_2_ → Cu_(3−x)_Ta_7_O_19_ + x CuO (surface) (x = 0 to ~0.8). With minor amounts of surface oxidation, the cathodic photocurrents of the polycrystalline films increase significantly, from <0.1 mA cm^−2^ up to >0.5 mA cm^−2^, under visible-light irradiation (pH = 6.3; irradiant powder density of ~500 mW cm^−2^) at an applied bias of −0.6 V vs. SCE. Electronic structure calculations revealed that its defect tolerance arises from the antibonding nature of its valence band edge, with the formation of defect states in resonance with the valence band, rather than as mid-gap states that function as recombination centers. Thus, the metastable Cu(I)-containing semiconductor was demonstrated to possess a high defect tolerance, which facilitates its high cathodic photocurrents.

## 1. Introduction

Recently, many mixed-metal oxide semiconductors containing a Cu(I) cation have emerged as promising *p*-type semiconducting photoelectrodes [1,2]. Incorporation of the Cu(I) cation within a metal-oxide semiconductor drives the formation of a higher-energy valence band edge, stemming from the 3*d*^10^ orbitals. This results in a shortened energetic distance to the conduction band of an early transition-metal or main group oxide, as found in several previous examples, such as CuRhO_2_ [3], Cu_2_WO_4_ [4], CuNbO_3_ [5], Cu_3_VO_4_ [6], and others. This often results in band gaps as low as ~1.5 to 2.5 eV in combination with an energetically-close alignment of the conduction band edge, with respect to the reduction potential of water and carbon dioxide. While only a few promising *p*-type semiconducting oxides have previously been reported, e.g., Cu_2_O [7,8] and CaFe_2_O_4_ [9,10], many new members of this emerging class of Cu(I)-containing oxides have formed p-type semiconductors. Thus, these new ternary Cu(I)-containing oxides represent an intriguing and quickly growing class of *p*-type semiconductors.

Low-temperature synthetic techniques, such as flux-mediated or hydrothermal [11,12,13], have been important for the preparation of many of the Cu(I)-containing ternary oxides. As has been recently reviewed [14], this occurs because when Cu_2_O is reacted with M_2_O_5_ (M = V, Nb or Ta) or M’O_2_ (M’ = Ti or Hf), nearly all of the ternary Cu(I)-containing oxides are thermodynamically unstable, i.e., metastable, with respect to decomposition to simpler oxides. For example, when Cu_2_Ta_4_O_11_ is heated in a vacuum, the following decomposition occurs: Cu_2_Ta_4_O_11_ → Cu_2_O + 2 Ta_2_O_5_. This reaction, and other alternative decomposition pathways, have been observed in all Cu(I)-containing niobates, tantalates, and vanadates [4,5,6,15,16,17,18]. For example, Cu_2_Ta_4_O_11_ is calculated to be metastable by ~0.04 eV atom^−1^, and thus its decomposition reaction is thermodynamically favorable. Most known Cu(I)-semiconductors are calculated to be metastable, as tabulated within the Materials Project database [19]. The relationship between their metastable character and their small visible-light bandgaps has been postulated to stem from their relatively weak Cu-O bonding and coordination number [14]. This leads to their relatively low formation energies as ternary oxides, when compared to a mixture of the simpler binary oxides that represent the thermodynamic ground state.

Cu(I)-containing semiconductors possess advantageously smaller band gaps (E_g_~1.3 to 2.6 eV) compared to most other metal oxides, with high attainable photocathodic currents (up to ~3–5 mA/cm^2^) in the form of polycrystalline films. The latter is enhanced by mild oxidation of their surfaces in air. For example, recent studies on several Cu-containing semiconductors, including CuNb_3_O_8_ [16,20], Cu_3_VO_4_ [6], Cu_5_Ta_11_O_30_ [17], and CuBi_2_O_4_ [21] have consistently shown that their decomposition and/or oxidation in air leads to the formation of CuO nano-islands on their surfaces. This has been found, in each case, to result in higher photocurrents, because of the increased charge separation efficiency arising from the type-II band alignments between the underlying semiconductor and the CuO islands at their surfaces. In addition, the slow extrusion of copper from the bulk structures yields surprisingly high concentrations of Cu-vacancy defects, which could potentially function as charge recombination centers. However, their relatively high photocurrents show their potential as a new class of defect-tolerant semiconductors. It is currently unclear what leads to this behavior for many Cu(I)-containing oxides, which is analogous to the high defect tolerance that is being intensely pursued in metal-halide semiconductors [22].

Presented herein is the flux synthesis and investigation of the photoelectrochemical properties of polycrystalline films of the metastable, Cu(I)-containing, Cu_3_Ta_7_O_19_. This p-type semiconductor was found to slightly oxidize at its surfaces, resulting in high cathodic photocurrents under visible-light irradiation. This occurred concomitantly with high concentrations of Cu-site vacancy defects (up to ~50%) and partial oxidation of the Cu(I) to Cu(II) cations, and yet with maintenance of its bulk crystalline structure. Its electronic structure was probed by density functional theory calculations, which revealed features that lead to its high defect tolerance. Thus, the metastable nature of this Cu(I)-containing semiconductor is demonstrated to be closely related to its ability to tolerate a high concentration of defect sites, leading to large cathodic photocurrents.

## 2. Results and Discussion

### 2.1. Bulk Phase Analysis and Thermal Stability

**Figure 1:**
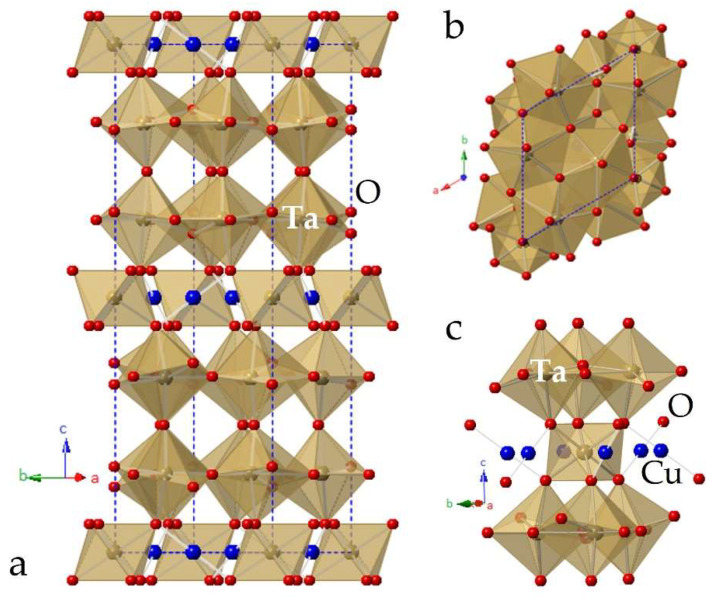
Structural views of Cu_3_Ta_7_O_19_ showing the (**a**) unit cell, (**b**) coordination geometry of the layers of TaO_7_ pentagonal bipyramids, and (**c**) coordination of the TaO_6_ octahedron and neighboring, linearly-coordinated Cu(I) cations. Atom types are labeled.

The powder XRD data, Figure S1 in the Supporting Information, show that the Cu_3_Ta_7_O_19_ compound was prepared with high purity using CuCl-flux mediated synthesis. The use of a CuCl flux is key to preparing this compound in high purity, as it is calculated to be metastable and to decompose to a mixture of Cu_2_O and Ta_2_O_5_ by 0.032 eV atom^−1^ [19]. A polyhedral view of its unit cell along the [110] crystallographic direction is shown in Figure 1. Briefly, Cu_3_Ta_7_O_19_ crystallizes in the hexagonal P6_3_/*m* space group, with a structure type that has been described previously [23]. The structure is comprised of layers of linearly-coordinated Cu(I) and octahedrally-coordinated Ta(V) cations that alternate with double layers of edge- and vertex-shared TaO_7_ pentagonal bipyramids. The layers of pentagonal bipyramids, i.e., the TaO_7_ in Figure 1b, are similar to those previously described in the structure of α-U_3_O_8_ [24].

**Figure 2:**
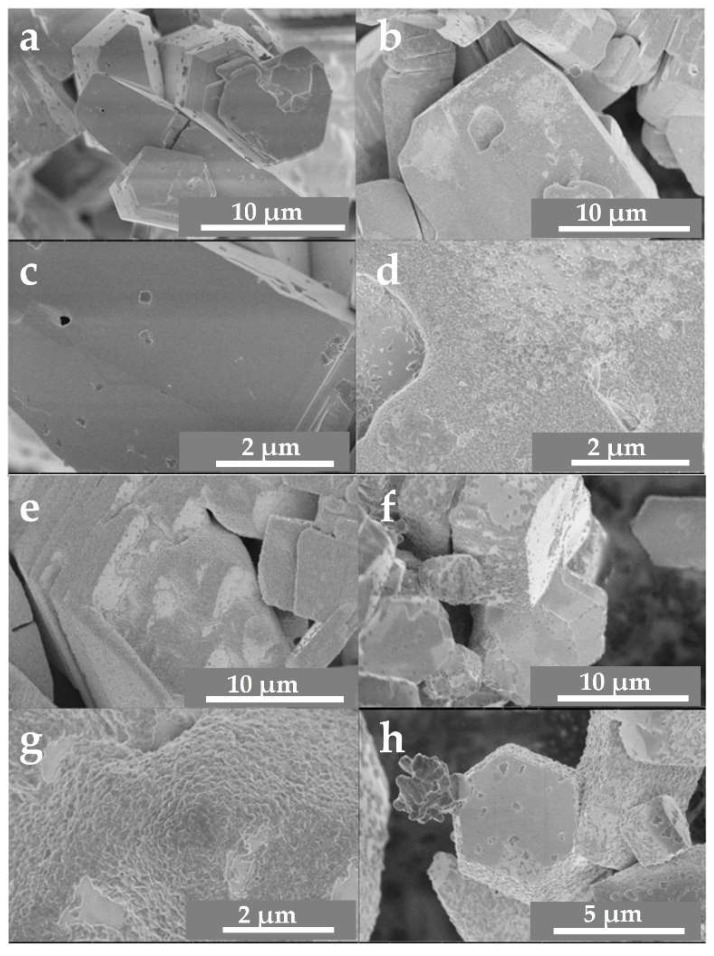
SEM images of Cu_3_Ta_7_O_19_ crystallites before heating (**a**,**c**), and after heating in air to 350 °C for 60 min (**b**,**d**), 450 °C for 60 min (**e**,**g**), and 550 °C for 60 min (**f**,**h**).

To probe the bulk and surface-level thermal stability of Cu_3_Ta_7_O_19_, the crystallites were heated in air at temperatures spanning the range of 350 °C to >550 °C. Shown in Figure 2a,c, scanning electron microscopy (SEM) images reveal micron-sized Cu_3_Ta_7_O_19_ crystallites with highly faceted and smooth surfaces, with a size distribution between ∼5 to 20 μm. After heating these in air at 350 °C for 60 min, 450 °C for 60 min, or at 550 °C for 60 min, increasing amounts of surface nano-islands were found, as shown in Figure 2. Interestingly, these surface nano-islands were only observed to form at the sides and stepped edges of the faces of the hexagonally-shaped particles. Powder X-ray diffraction (PXRD) data, see the Supporting Information, show that these corresponded to growing amounts of surface oxidation of Cu(I) to Cu(II) cations and a subsequent phase segregation at the surfaces. These results are consistent with a similar surface-mediated oxidation pathway as found previously for CuNb_3_O_8_, Cu_3_VO_4_, Cu_5_Ta_11_O_30_, and Cu_2_WO_4_ [4,6,16,17]; i.e., heat treatments in air of these Cu(I)-containing semiconductors result in their surface oxidation, with the formation of CuO nano-islands. The preferential growth of the nano-islands over the edges arises because of the facile diffusion path of copper cations within the layers that are aligned with the *ab*-plane of the structure, as investigated by electron microscopy and described previously for the related Cu_5_Ta_11_O_30_, which contains structurally similar layers of pentagonal bipyramids [17].

**Figure 3:**
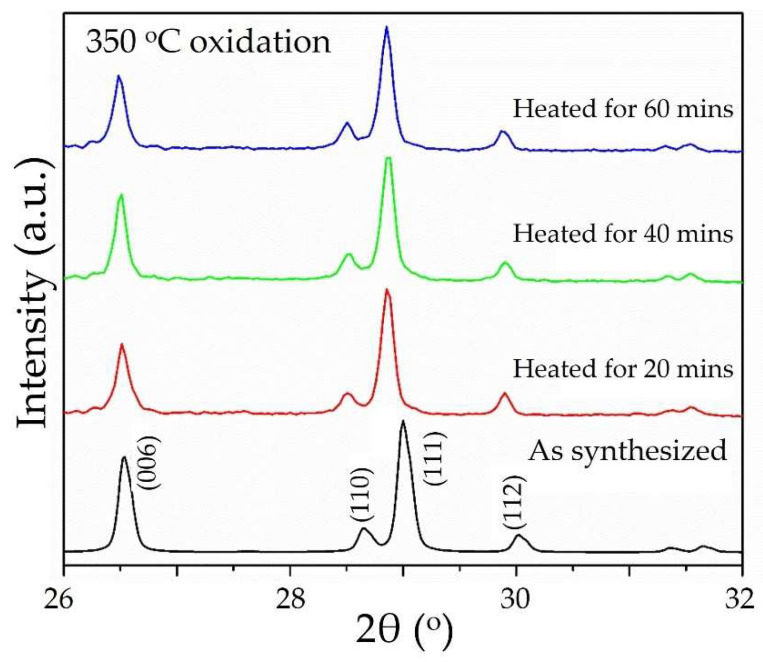
Powder XRD data, illustrating the peak shiting after heating Cu_3_Ta_7_O_19_ in air to 350 °C for 20 min, 40 min, and 60 min.

Refinements of the unit cell or full crystalline structure of Cu_3_Ta_7_O_19_ were performed after heating in air, in order to understand the bulk changes in the crystalline structure that accompany the surface oxidation. Shown in Figure 3, and in Table S2 in the Supporting Information, the XRD peaks shifted to increasingly smaller 2θ° angles with increasing reaction temperature and reaction time, yielding a larger unit cell, by up to ~10% at 550 °C for 60 min. For a sample heated to 350 °C for 60 min, a full structure Rietveld refinement shows the crystalline structure is maintained with a composition of Cu_(3−*x*)_Ta_7_O_19_ (*x* = 0.34(1)), or with a copper-deficiency of ~11.3%, as listed in Table S1 and shown in Figure S2. By analogy, other Cu(I)-containing tantalates in this family occur naturally with large amounts of Cu(I)-vacancies (for charge balancing reasons in their ‘idealized’ structures), including Cu_2_Ta_4_O_11_ (33% vacancies) and Cu_5_Ta_11_O_30_ (16.6% vacancies) [23,25]. Thus, while the ideal crystal structure contains 0% vacancies, upon mild oxidation it appears to be capable of sustaining a crystalline structure with large amounts of Cu(I)-vacancies.

The ion diffusion and phase segregation of Cu(I) cations out of the structure, and their oxidation to CuO at the surfaces, can be represented by the following reaction: Cu_3_Ta_7_O_19_ + x/2 O_2_ → Cu_(3−x)_Ta_7_O_19_ + x CuO(surface). With the extrusion of Cu(I) cations from the structure and oxidation at the surfaces, an equal number of Cu sites within the bulk are also oxidized, as given by simple charge balancing: Cu^I^_(3–2x)_Cu^II^_x_Ta_7_O_19_. Temperature-dependent magnetic susceptibility measurements were taken on Cu_3_Ta_7_O_19_ before and after heating in air from 250 °C to 450 °C. Listed in Table S3, the data were fitted to the Curie–Weiss expression (Cu(II) of S = ½) with the refined parameters. Upon heating in air, the effective magnetic moment increased from ~0.179μ_B_ with no heating, to 0.216μ_B_, 0.452μ_B_, and 0.917μ_B_ after heating in air to 250 °C, 350 °C, and 450 °C, respectively. Accounting for both the surface and bulk Cu(II) cations, these values correspond to %Cu(II) cations (i.e., x) in the bulk Cu_(3−x)_Ta_7_O_19_, ranging from ~10.4% (x = 0.015) to 26.1% (x = 0.39) at 50 °C, and to 53.0% (x = 0.8), respectively. This analysis is consistent with the refined Cu-site vacancy from the Rietveld analysis, with x = 0.34(1) after heating to 350 °C in air, and supports the oxidation of Cu(I) to Cu(II) cations within the structure and at its surfaces according to the above reaction. Full oxidation eventually leads to CuTa_2_O_6_, as shown in the powder XRD data in Figure S3.

### 2.2. Optical and Photoelectrochemical Properties

**Figure 4:**
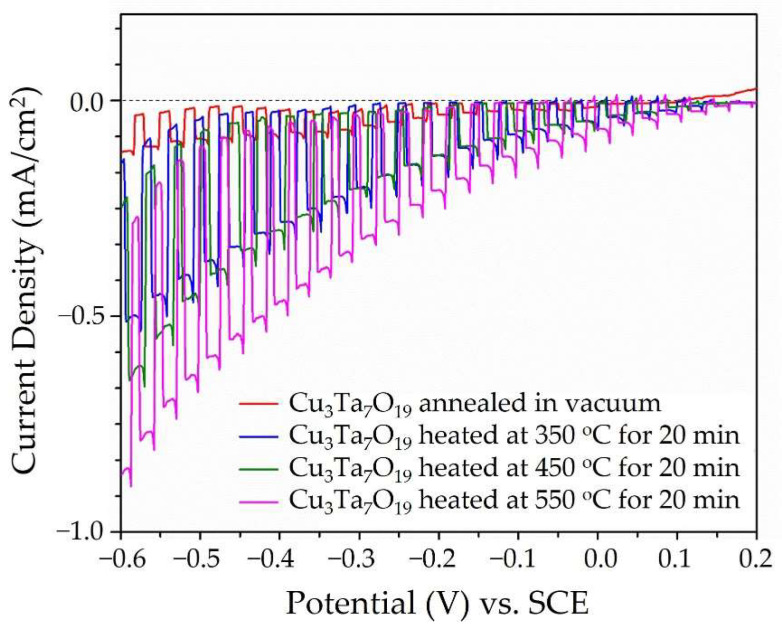
Current density as a function of applied potential versus the standard calomel electrode (SCE) in an aqueous 0.5M Na_2_SO_4_ solution at pH ~ 6.3 under chopped visible light irradiation for Cu_3_Ta_7_O_19_ films annealed in a vacuum at 500 °C, and after heating in air for 20 min at 350 °C (blue), 450 °C (green), and at 550 °C (magenta).

**Figure 5:**
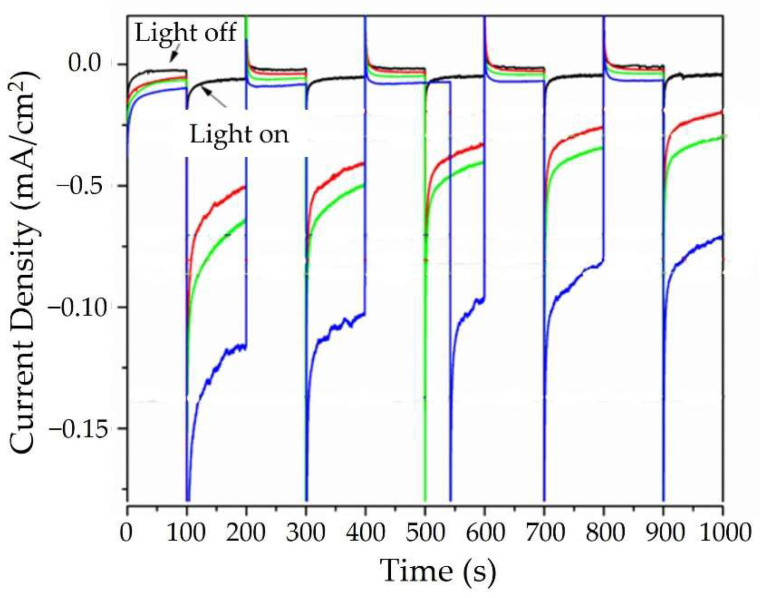
Chronoamperometry (−0.25 V applied bias versus SCE) in aqueous 0.5 M Na_2_SO_4_ solution at pH ~ 6.3 under chopped visible light irradiation for Cu_3_Ta_7_O_19_ films annealed in a vacuum at 500 °C (black), and after heating in air for 20 min at 350 °C (red), 450 °C (green), and at 550 °C (blue).

Tauc plots of the UV-Vis diffuse reflectance data show that Cu_3_Ta_7_O_19_ had a band gap of ~2.5 eV, Figure S4, consistent with earlier reports [23] and the bright yellow color of its powder. Polycrystalline films of the Cu_3_Ta_7_O_19_ semiconductor were deposited onto FTO slides using a previously reported drop-cast method and annealed in vacuum at 500 °C. All films showed a p-type semiconducting behavior, with an increasing photocathodic current with negative applied biases. Higher photocurrents were found for films that had been heated in air at increasing temperatures to slightly oxidize their surface. Shown in Figure 4, the non-oxidized films showed the lowest photocurrent density (red curve; ~0.1 mA cm^−2^), and this increased after heating at 350–550 °C in air for 20 min (blue, green, and purple curves; ~0.3–0.6 mA cm^−2^ after subtracting the dark current) under chopped visible light irradiation at 500 mW cm^−2^ and pH = 6.3. Chronoamperometry data taken at −0.25 applied bias, Figure 5, revealed a decay in the photocurrent densities across all films of ~33% after ~17 min, as a result of the deactivation of the surfaces. The highest photocathodic current was found after heating at 550 °C in air for Cu_(3−x)_Ta_7_O_19_, corresponding to the composition with the highest number of Cu-site vacancies. The polycrystalline film of Cu_3_Ta_7_O_19_ was also heated to 450 °C at increasing time intervals of 20, 40, and 60 min, to investigate the impact of increasing thermal oxidation. The photocathodic currents ranged from <0.1 mA cm^−2^ at 0 V applied bias to ~1.0 mA cm^−2^ at −0.6 applied bias, and generally increased by ~50% after heating from 20 min to 60 min, as shown in Figures S5 and S6 in the Supporting Information. Powder X-ray data were taken on the films after the photoelectrochemical measurements and showed no evidence of bulk changes.

**Figure 6:**
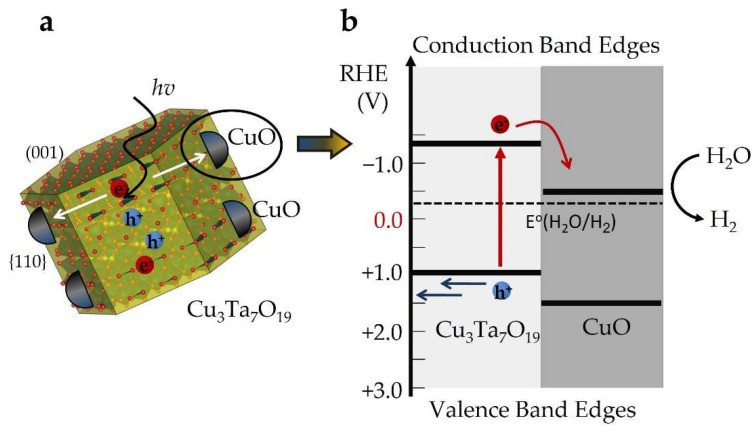
(**a**) Polyhedral model of a Cu_3_Ta_7_O_19_ crystallite with CuO surface islands on its {110} faces, and (**b**) alignment of their conduction and valence-band edge energies at the interface, with a band-offset favorable for H_2_O reduction at the surfaces at pH ~ 6.3.

The oxidation of Cu(I) to Cu(II), occurring both at the surface and in the bulk, yielded much higher photocurrents for the Cu_(3−x)_Ta_7_O_19_ films. This result is surprising given the high concentration of Cu-site vacancies, of up to 50%, that formed when heating in air at 450 to 550 °C. Prior work on Cu(I)-containing semiconductors has shown that the phase segregation of CuO at the surface can result in a favorable band-energy offset to drive more efficient charge separation, such as in Cu_(3−x)_VO_4_ and CuBi_2_O_4_ [6,21]. For p-type Cu_3_Ta_7_O_19_, its band edge energies were determined from Mott–Schottky measurements that yielded a flat band potential of +0.36 V (versus SCE at pH = 6.3). This gave band edge energies of −1.57 V and +0.73 V for the conduction and valence band edges, respectively. The conduction band edge is therefore located at a significantly more negative potential than for CuO, which occurs at about −0.8 V (+/− 0.1 V) at pH ~ 6.0 versus SCE [16,26]. The offset of the conduction band edges thus facilitates the diffusion of minority carriers from p-type Cu_3_Ta_7_O_19_ to CuO, followed by the reduction of protons to dihydrogen at the surfaces of the latter, as illustrated in Figure 6. Conversely, the valence band edge of Cu_3_Ta_7_O_19_ is located at a more negative potential than in CuO, inhibiting the concomitant hole migration to the surfaces of CuO. Thus, a favorable type-II band offset is formed between p-type CuO and Cu_3_Ta_7_O_19_, as shown in Figure 6, yielding a net diffusion of electrons to the surfaces and an increased charge separation efficiency.

### 2.3. Electronic Structure and Defect Tolerance

The increased photocurrent with higher concentrations of Cu(I)-vacancies in Cu_3_Ta_7_O_19_ is perhaps surprising, given the potential impact of defect sites functioning as charge recombination centers. In semiconductors, defects can function as deleteroius recombination centers if they introduce mid-gap states within the electronic structure. However, if the formation of mid-gap states can be avoided or suppressed, then the semiconductor is described as being ‘tolerant’ to the formation of defects and can still function as an efficient photoelectrode [22]. It is unclear whether the common occurrence of Cu(I)-site vacancies acts to introduce mid-gap states in this class of p-type semiconductors.

**Figure 7:**
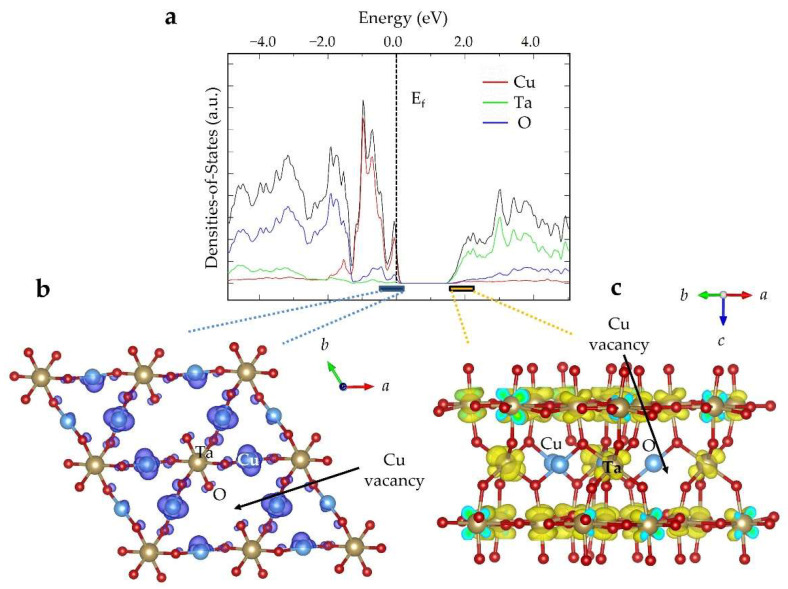
Cu_(3−x)_Ta_7_O_19_ (x = 0.33), the (**a**) calculated densities-of-states, with the atomic orbital contributions and Fermi level (E_f_) labeled, and the electron density contributions to the (**b**) valence band edge (blue bar, colored) and (**c**) conduction band edge (yellow bar, colored).

In Cu_(3−x)_Ta_7_O_19_, Cu-site vacancy defects are shown to occur in the bulk (up to x = 0.8) as a result of their extrusion from the structure and oxidation at the surfaces to CuO. Electronic structure calculations were performed using density-functional theory methods, to investigate the energies and impact of the Cu-site vacancies, as well as the atomic contributions to the conduction and valence band edges, as illustrated in Figure 7 for the structure of Cu_(3−x)_Ta_7_O_19_ with 8.3% Cu-site vacancies, i.e., x = 0.33. The electron densities at the conduction and valence band edges are shown to stem from the filled Cu 3d^10^ and empty Ta 5d^0^ orbitals, respectively, as found previously for the idealized structure without Cu-site vacancies [27]. The introduction of Cu-site vacancies, however, has shifted the Femi level into the valence band maximum, Figure 7a, with the partial depopulation of the Cu-based 3d^10^ orbitals. This is consistent with the partial oxidation of Cu(I) to Cu(II) cations, as was found experimentally. The electron densities at the conduction and valence band edges remain unchanged, as a result of the introduction of Cu-site vacancies shown in Figure 7b,c, and, furthermore, no new energetic states were introduced within the bandgap. Rather, the new energetic states stemming from the Cu-site vacancies stay in resonance with the valence band, as is required for semiconductors in order to exhibit defect tolerance. This situation arises because of the antibonding nature of the Cu-O (d-to-p σ*-type) interactions at the valence band maximum in the electronic structure, Figure 7b. The formation of a Cu-site vacancy therefore lowers the O 2p orbital energies in resonance with the valence band, instead of raising them and forming a mid-gap energetic state. Thus, the Cu_(3−x)_Ta_7_O_19_ structure shows a high defect tolerance, with its high concentrations of Cu-site vacancies yielding increasing cathodic photocurrents, due to the formation of CuO at the surfaces.

## 3. Conclusions

A metastable p-type Cu_3_Ta_7_O_19_ semiconductor was prepared by a flux-mediated synthetic route and investigated for its photoelectrochemical properties. Upon heating in air, the Cu(I) cations slowly oxidized to Cu(II) at the surfaces and in the bulk, as probed by electron microscopy and magnetic susceptibility measurements. In the form of polycrystalline films, increasing cathodic photocurrents of up to >0.5 mA cm^−2^ (under visible-light irradiation) are observed after heating in air from 350 °C to 550 °C, from 20 to 60 min. These increased photocurrents originate from the formation of a type-II band offset between Cu_(3−x)_Ta_7_O_19_ and CuO at the surfaces, which can facilitate an enhanced charge separation. Furthermore, electronic structure calculations show that these high photocurrents were facilitated by the defect tolerance of the structure for Cu-site vacancies and the absence of mid-gap energetic states, which could potentially function as recombination centers.

## 4. Materials and Methods

### 4.1. Materials

The reactants were reagent grade and used as received for Ta_2_O_5_ (Alfa Aesar, min 99.99%), Cu_2_O (Alfa Aesar, 99.99%), and CuCl (Alfa Aesar, 99.99%). For the photoelectrochemical measurements, the reagents Na_2_SO_4_ (Alfa Aesar, 99.0%), NaOH (>97%, Fisher Scientific), and deionized water were also used.

### 4.2. Flux Synthesis

A flux-mediated synthetic approach was used for the preparation of Cu_3_Ta_7_O_19_, with the low melting CuCl (m.p. = 426 °C) salt as the flux. Stoichiometric amounts of Cu_2_O and Ta_2_O_5_ were finely ground together within a mortar and pestle for 30 min inside an Ar-filled glovebox. To this, the CuCl salt flux was added in a 10:1 molar ratio of salt:product and the powder was loaded into a previously cleaned quartz tube. After sealing under vacuum, the reaction vessel was heated inside a muffle furnace to 800 °C for 26 h and allowed to radiatively cool to room temperature. The products were washed with 3M NH_4_OH in order to remove the CuCl flux, and yielding a bright, yellow-colored powder that was determined by powder X-ray diffraction to be Cu_3_Ta_7_O_19_ in high purity. To investigate the decomposition of Cu_3_Ta_7_O_19_ upon heating, samples of the high-purity powder were placed into an alumina crucible and heated in air in a muffle furnace in intervals from 350 °C up to 750 °C for heating times from 20 min to 3 h.

### 4.3. Characterization

An Inel X-ray diffractometer was used to take powder X-ray diffraction (XRD) data, with Cu Kα_1_ radiation (λ = 1.54056 Å) generated from a sealed tube X-ray generator (30 mA, 35 kV). A sample mass of ~50 mg was dispersed between two pieces of Scotch tape and fixed into a rotating sample holder. A curved position sensitive detector was used in transmission mode to measure the XRD diffractograms, with exposure times from 30 to 90 min. The lattice constants of the bulk powders were refined using the Rietveld method, using whole pattern fitting within Jade 9 software [28]. The unit cell dimensions and volumes are provided in Tables S1 and S2 in the Supporting Information, for the as-synthesized Cu_3_Ta_7_O_19_ and for the product after it was heated in air, in intervals from 350 °C to 750 °C.

A full structural refinement of the Cu_3_Ta_7_O_19_ compound was performed after oxidation in air at 350 °C for 60 min. Powder XRD data were collected on a Philips X-pert diffractometer using Cu Kα radiation at room temperature. An angular range of 10° ≤ 2θ ≤ 110° was collected with a step width of 0.017°. The structural refinement proceeded by the Rietveld method within the JANA2000 software package [29]. A Legendre polynomial containing 15 coefficients was used to estimate the background. Bragg peak shapes were modeled using five profile coefficients of a pseudo-Voigt function. The starting structural parameters were taken from the crystalline structure of Cu_3_Ta_7_O_19_ [23], crystallizing in the space group P6_3_/*m* (no. 176) with lattice constants *a* = 6.2278(1) Å and *c* = 20.1467(3) Å and *V* = 672.7(1) Å^3^. Its crystalline structure consists of two symmetry-unique Ta sites, one Cu site, and five oxygen sites. Refinement of the background and the peak shape parameters proceeded first, followed by zero shift parameter and the dimensions of the unit cell, refining to *a* = 6.2403(1) Å and *c* = 20.0835(3) Å and *V* = 677.30(2) Å^3^. The atomic coordinates were refined next, starting with the heavier Ta and Cu atoms, and then the O atoms. Lastly, the Cu-site occupation was allowed to refine and it converged to an occupancy of 0.887, giving a refined composition of Cu_2_._67_Ta_7_O_19_ that is consistent with the contracted unit cell volume (by −0.06%) and ~11% Cu-site vacancies. The final refined *R* and *R_wp_* factors converged to 5.61% and 9.45%, respectively. Selected refinement parameters and interatomic distances are given in the Supporting Information.

UV-Vis diffuse reflectance spectra were collected using an integrating sphere within the wavelength range of 200 nm to 1300 nm. A pressed BaSO_4_ powder was used as the reference background. In a typical measurement, ~30 mg of powder was mixed with the BaSO_4_ reference powder and mounted onto a flat sample holder. The holder was then placed into the external window of the integrating sphere. As described previously [17], the reflectance data were plotted as Tauc plots of (*F*(*R*)*hν*)*^n^* vs. (*hν*) to estimate the direct (*n* = ½) and indirect (*n* = 2) bandgap energies. Scanning electron microscopy (SEM) images were taken on a field-emission scanning electron microscope to characterize the particle morphologies, surfaces features, and qualitative chemical compositions. The thermal stability and reactivity of the samples were measured as a function of temperature and the data were analyzed as the % weight change of the sample versus temperature.

### 4.4. Photoelectrochemical Measurements

Polycrystalline films of Cu_3_Ta_7_O_19_ were prepared on TEK-15 fluorine-doped tin oxide (FTO) slides. The slides were first sonicated in deionized water, followed by ethanol and acetone for 30 min each. On the conducting side, an ~1 cm^2^ area was taped off using Scotch 3M tape and powdered samples were deposited using drop casting and doctor blade techniques, according to previous reports [16,17]. A water/tert-butanol solution was used as a medium for prepare the dispersion of the powder. The drop-cast films were annealed at 500 °C for 3 h under a dynamic vacuum. The films were then used heated in air for slow surface oxidation at temperatures of 350 °C, 450 °C, and 550 °C, for a heating time of 20 min each. The polycrystalline films were mounted into a custom-built photoelectrochemical cell for measurements of the photocurrent densities within a 0.5M Na_2_SO_4_ solution as electrolyte at a pH of ~6.3. Linear-sweep cyclic voltammograms were taken at an applied bias range of 0.2V to −0.6V under chopped irradiation from a high pressure Xe lamp for each film. The arc lamp was equipped with infrared and ultraviolet photon cutoff filters, to give an output wavelength range of 1000 nm to 420 nm at an irradiant power density of ∼500 mW/cm^2^. Chronoamperometric measurements were also taken at a constant applied bias of −0.25 V, as a gauge of the photocurrent decay versus time.

### 4.5. Electronic Structure Calculations

Plane-wave density functional theory was utilized to perform electronic band-structure calculations on the geometry-optimized and Cu-deficient Cu_(3−x)_Ta_7_O_19_ structure, as implemented in the *Vienna Ab-Initio Simulation Package* (VASP; ver. 4.6) [30]. The refined crystal structures of Cu_2_._67_Ta_7_O_19_ were utilized in the calculations. A Cu-site vacancy concentration of ~8.3% was modeled using a 2 × 2 × 1 superstructure, which enabled a random distribution of 1 Cu-site vacancy per four unit cells of Cu_3_Ta_7_O_19_. Perdew–Burke–Ernzerhof functionals were employed in the calculations within the generalized gradient approximation [31]. The Monkhorst–Pack scheme was used for automatic selection of 36 *k*-points within the Brillouin zone [32].

## Data Availability

Data supporting the findings of this study are available from the corresponding author (P.A.M.) on request.

## Supplementary Materials

Figure S1–S3: Powder XRD data for as-synthesized Cu_3_Ta_7_O_19_ and after heating in air, Table S1: Selected Rietveld refinement parameters, Table S2: Refined unit cell dimensions and unit cell volume, Figure S4: Tauc plots of UV-Vis diffuse reflectance data, Figure S5: Current density versus applied potential for polycrystalline films heated to 450 °C for 20 min, 40 min, and 60 min, under visible-light irradiation, Figure S6: Chronoamperometry of the polycrystalline films heated to 450 °C, Table S3: Refined Curie–Weiss parameters from temperature-dependent magnetic susceptibility data.

## Sample Availability

Samples of the compounds are available from the authors.
