# Supplementary material for: A Metastable p-Type Semiconductor as a Defect-Tolerant Photoelectrode
Source: Molecules. 2021 Nov 12;26(22):6830. doi: 10.3390/molecules26226830 (PMC8624039; doi:10.3390/molecules26226830)
Supplement: Supplementary file 1 [file molecules-26-06830-s001.zip › molecules-1443944-supplementary.pdf]

Supporting Information

"A Metastable p-Type Semiconductor as a Defect-Tolerant Photoelectrode"

Zahirul Sohag, Shuan O'Donnell, Lindsay Fuoco and Paul A. Maggard *
Department of Chemistry, North Carolina State University, Raleigh, NC 27609-8204

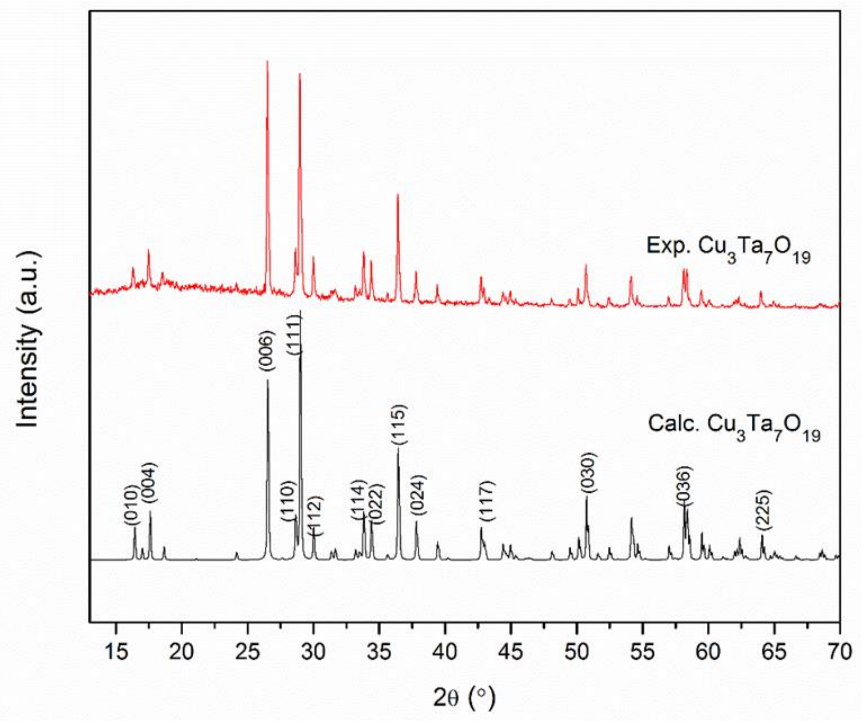

Figure S1. Powder X-ray diffraction pattern of the as-synthesized $Cu_3Ta_7O_{19}$ (upper) and of the calculated theoretical pattern (lower).

Table S1. Selected Rietveld refinement parameters for $Cu_{2.66(1)}Ta_7O_{19}$.

Formula	$Cu_{2.66(1)}Ta_7O_{19}$
Formula weight (g/mol)	1739.8
Space group, Z	P$6_3/m$
Radiation	Cu Kα_1= 1.54051 Å<br>Kα_2= 1.54433 Å
Crystal system	Hexagonal
a/Å	6.2403(1)
c/Å	20.0835(3)
Volume/Å^3	677.30(2)
R_p, R_{wp}	0.0561, 0.0945
2θ (°) range	10–110
No. of unique data	5834

Table S2. Refined unit cell dimensions and volume for $Cu_3Ta_7O_{19}$ after heating in air.

Time (min), 450 °C in Air	*a* (Å)	*c* (Å)	Volume (Å^3)	Δ Volume
0	6.226(1)	20.159(7)	676.766	0
20	6.253(1)	20.120(7)	681.469	+4.69
40	6.257(2)	20.15(1)	683.360	+6.59
60	6.261(1)	20.161(9)	684.617	+7.84
**Time (min), 550 °C in Air**	***a* (Å)**	***c* (Å)**	**Volume (Å^3)**	**Δ Volume**
0	6.226(1)	20.159(7)	676.766	0
20	6.256(1)	20.055(6)	679.779	+3.00
40	6.26(1)	20.13(5)	684.780	+8.00
60	6.27(1)	20.13(6)	686.770	+9.99

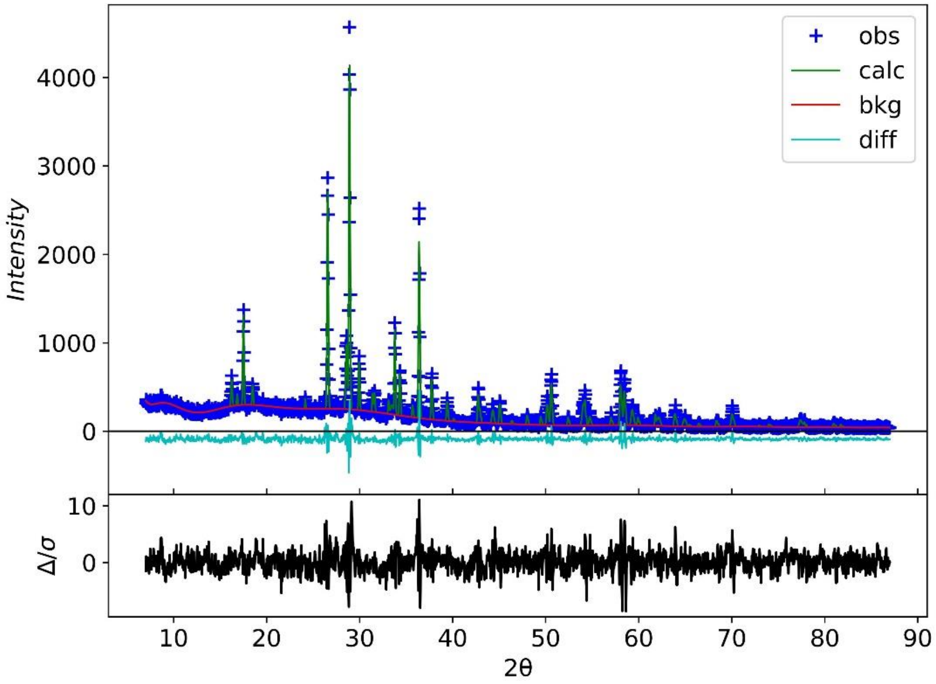

Figure S2. Rietveld refinement of $Cu_{2.66(1)}Ta_7O_{19}$ after heating to 350 ºC for 60 min.

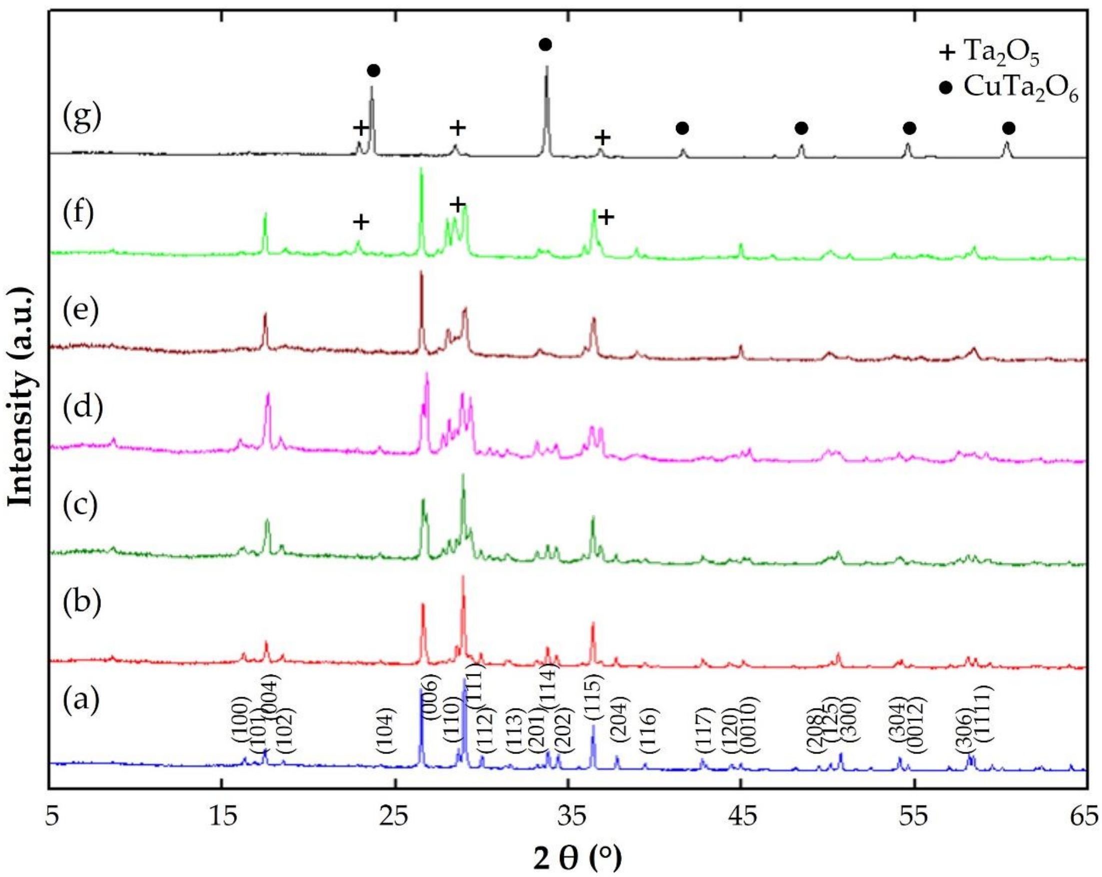

Figure S3. Powder X-ray diffraction patterns for $Cu_3Ta_7O_{19}$ before heating (a), and after heating in air at 400 °C for 3 h (b), 400°C for 12 h (c), 450°C for 12 h (d), 550°C for 3 h (e), 650 °C for 3 h (f) and 750°C for 3 h (g).

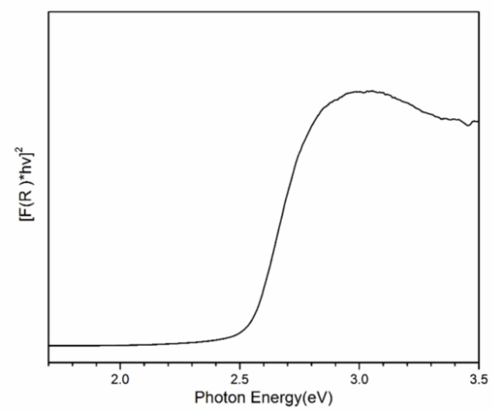

(left)

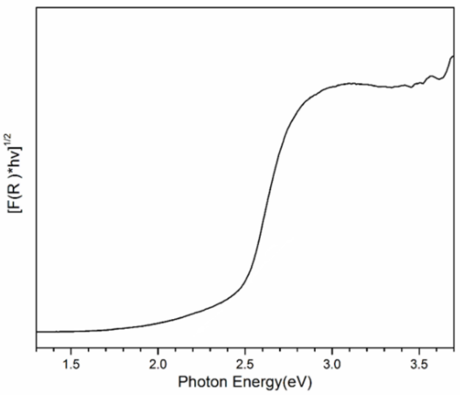

(**right**)

Figure S4. Tauc plots for $Cu_3Ta_7O_{19}$ for the direct bandgap transition **(left**) and indirect bandgap transition (**right**).

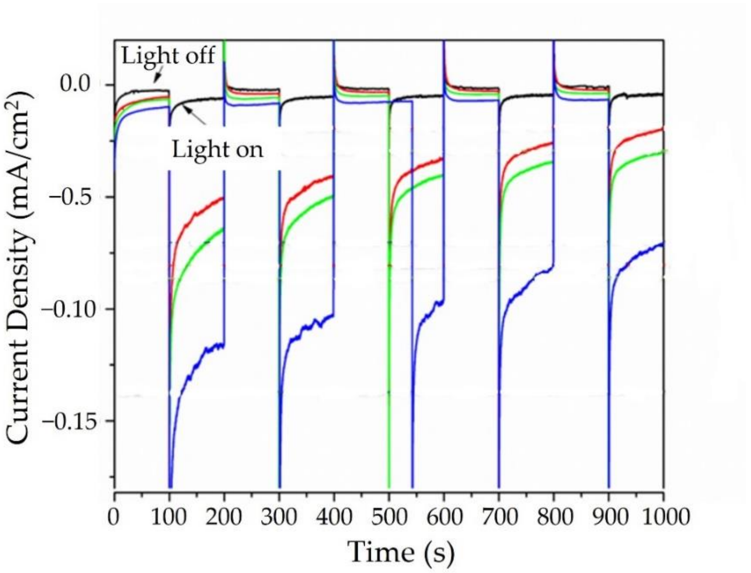

Figure S5. Current density versus applied potential in an aqueous 0.5M Na_2SO_4 solution at pH ~6.3 under chopped visible light irradiation for $Cu_3Ta_7O_{19}$ films annealed in vacuum at 500 °C, and after heating in air at 450 ^oC for 20 min (red), 40 min (green) and 60 min (blue).

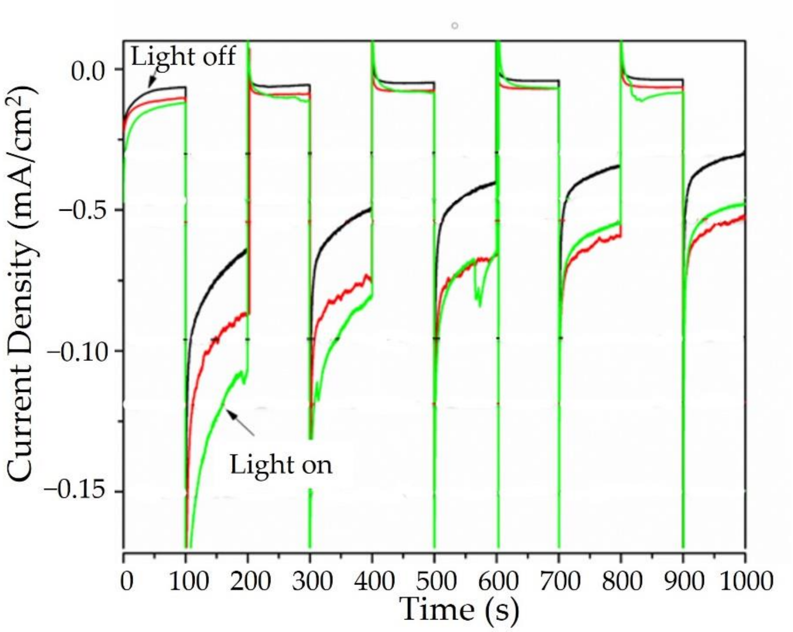

Figure S6. Chronoamperometry (−0.25V applied bias) in aqueous 0.5M Na_2SO_4 solution at pH ~6.3 under chopped visible light irradiation for $Cu_3Ta_7O_{19}$ films annealed in vacuum at 500 °C (black), and after heating in air at 450 ^oC for 20 min (black) 40 min (red) and 60 min (green).

Table S3. Refined Curie-Weiss parameters from temperature-dependent magnetic susceptibility data.

Reaction Conditions	ϴ (K)	C (emu K/mol)	TIP (emu/mol)	R^2	μ_{eff} per Cu	%Cu(II)
None	−3.908	0.0361	6.1×10^{-5} (75–100 K)	0.9784	0.1790	10.35
250 °C	8.016	0.0524	3.4×10^{-4} (200–300 K)	0.9612	0.2157	12.47
350 °C	−1.363	0.2298	8.6×10^{-4} (65–200 K)	0.9993	0.4519	26.12
450 °C	−1.270	0.9468	3.7×10^{-4} (50–300 K)	0.9996	0.917	53.03
